# Neuronal activity in the ventral tegmental area during goal-directed navigation recorded by low-curvature microelectrode arrays

**DOI:** 10.1038/s41378-024-00778-2

**Published:** 2024-10-14

**Authors:** Wei Xu, Mixia Wang, Gucheng Yang, Fan Mo, Yaoyao Liu, Jin Shan, Luyi Jing, Ming Li, Juntao Liu, Shiya Lv, Yiming Duan, Meiqi Han, Zhaojie Xu, Yilin Song, Xinxia Cai

**Affiliations:** 1 State Key Laboratory of Transducer Technology, Aerospace Information Research Institute, Chinese Academy of Sciences, Beijing, 100190 China; 2 School of Electronic, Electrical and Communication Engineering, University of Chinese Academy of Sciences, Beijing, 100049 China

**Keywords:** Nanoparticles, Electrical and electronic engineering, Biosensors

## Abstract

Navigating toward destinations with rewards is a common behavior among animals. The ventral tegmental area (VTA) has been shown to be responsible for reward coding and reward cue learning, and its response to other variables, such as kinematics, has also been increasingly studied. These findings suggest a potential relationship between animal navigation behavior and VTA activity. However, the deep location and small volume of the VTA pose significant challenges to the precision of electrode implantation, increasing the uncertainty of measurement results during animal navigation and thus limiting research on the role of the VTA in goal-directed navigation. To address this gap, we innovatively designed and fabricated low-curvature microelectrode arrays (MEAs) via a novel backside dry etching technique to release residual stress. Histological verification confirmed that low-curvature MEAs indeed improved electrode implantation precision. These low-curvature MEAs were subsequently implanted into the VTA of the rats to observe their electrophysiological activity in a freely chosen modified T-maze. The results of the behavioral experiments revealed that the rats could quickly learn the reward probability corresponding to the left and right paths and that VTA neurons were deeply involved in goal-directed navigation. Compared with those in no-reward trials, VTA neurons in reward trials presented a significantly greater firing rate and larger local field potential (LFP) amplitude during the reward-consuming period. Notably, we discovered place fields mapped by VTA neurons, which disappeared or were reconstructed with changes in the path–outcome relationship. These results provide new insights into the VTA and its role in goal-directed navigation. Our designed and fabricated low-curvature microelectrode arrays can serve as a new device for precise deep brain implantation in the future.

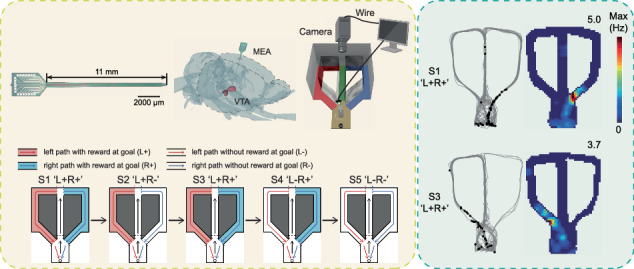

## Introduction

Navigating a goal position in response to environmental changes is crucial for survival. Goal positions typically contain reward resources, such as food and shelter. Animals must develop memories and associations with newly emerging reward positions and pathways while reducing associations with locations and pathways that no longer contain rewards, thereby increasing the probability of foraging success and reducing energy expenditure. Numerous brain regions are involved in goal-directed navigation, including the hippocampus, entorhinal cortex, prefrontal cortex, and basal ganglia, each of which play varying roles in spatial navigation, experiential learning, and memory^1–3^.

The ventral tegmental area (VTA) is located in the basal ganglia and is primarily composed of dopaminergic neurons, GABAergic neurons, and glutamatergic neurons^4–6^. Previous evidence has revealed that the VTA plays a crucial role in reward coding. Dopaminergic neurons in the VTA represent the difference between expected and actual rewards (reward prediction error, RPE)^7–9^. Based on RPE signals, various reinforcement learning (RL) models have been developed to explain the activity of dopamine neurons^10,11^. Many recent studies have focused on the VTA response during goal-directed navigation. VTA neurons are not only related to reward coding but also play significant roles in motivation, kinematics, and cognition^12–15^. This may be related to the extensive projection relationship between the upstream and downstream regions of the VTA^16–18^, which has excitatory (dopaminergic) or inhibitory (GABAergic) neural pathways with many regions, such as the striatum, nucleus accumbens, hippocampus, and prefrontal cortex^6,17,19^. Currently, the role of VTA neurons in goal-directed navigation needs to be further explored, particularly with respect to how rats learn and adapt to rapidly changing environmental factors in a short period.

Currently, many researchers use classical microwires or tetrodes to detect VTA neurons^20^, which are convenient to prepare but pose challenges in improving spatial resolution, reducing implant damage, and increasing the number of channels. In contrast, silicon-based implantable microelectrode arrays (MEAs) based on standard micro-electromechanical system (MEMS) processes offer the advantages of high resolution^21^ and a lower Young’s modulus (Si, 169 GPa) than the tungsten-based tetrode electrodes^22^ (tungsten, 400 GPa), and they facilitate an increase in electrode channels. Since MEA is a composite membrane structure formed by the deposition of different films at high temperatures, residual stresses are usually generated on it. The residual stress of multilayer films often affects device performance^23,24^. For long MEA probes, the presence of residual stress causes the electrode shank (implantable part) to bend and become prone to breakage. The phenomenon of electrode bending occurs in both rigid (such as silicon-based Neuropixels^25,26^ and silicon carbide (SiC)^27^) and flexible^28,29^ electrodes. Given the deep and small spatial location of the VTA in the brain, the curvature of the electrode shank caused by residual stress affects spatial implantation accuracy and can cause unnecessary damage to brain tissue during the surgical procedure.

In this study, we designed and fabricated silicon-based MEAs with high temporal and spatial resolution for detecting VTA neuronal signals in rats. Thereafter, we used the backside dry etching method to successfully release residual stress, ensuring that the electrode shank remained straight. Subsequently, platinum nanoparticles (PtNPs) were modified on the electrode site surface to improve the electrical properties of the MEAs while maintaining biocompatibility^30,31^. The rats participated in spatial navigation within a free-choice T-maze with successively changing path–outcome relationships. By combining the behavior of the rats in the experiment with the electrophysiological signal of VTA neurons, we analyzed neuronal activity across sessions in both the temporal and spatial domains, revealing the rapid response to reward and the place field remapping induced by reward within VTA neurons.

## Materials and methods

### Fabrication of the MEAs

A 16-channel microelectrode array targeting the VTA was designed. The total length of the MEA device was 14 mm, and its thickness was 27.1 μm. The electrode shank measured 11 mm in length and 280 μm in width.

**Fig. 1:**
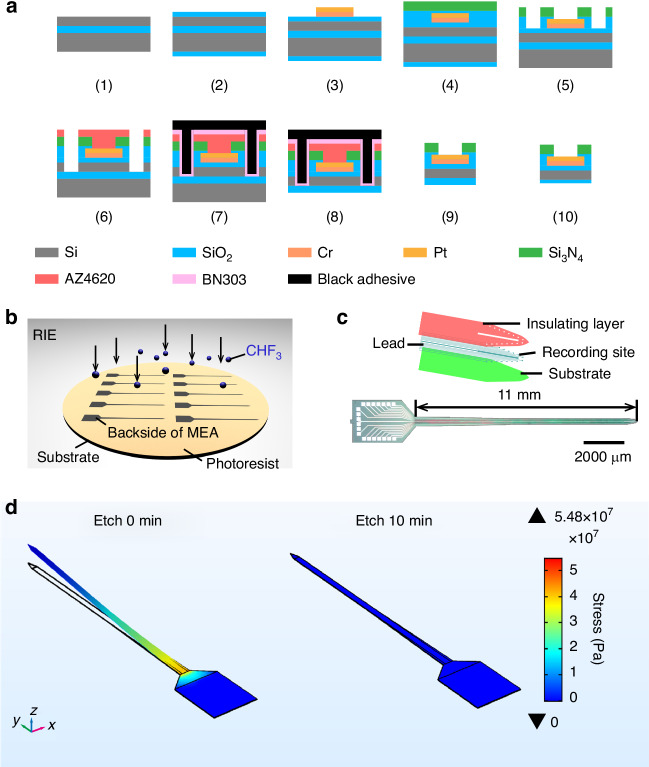
Fabrication and residual stress relief of the MEA. **a** Manufacturing process of the MEA. (1) Clean the SOI (silicon on insulator). (2) Thermally oxidize the substrate to form the insulating layer. (3) Sputter the metal layer. (4) Deposit SiO_2_/Si_3_N_4_ insulating layers via plasma-enhanced chemical vapor deposition (PECVD). (5) Etch the insulating layer to expose the electrode sites. (6) Perform deep silicon etching to define the electrode morphology. (7) Coat the front side of the SOI with black adhesive. (8) Wet etch the backside Si layer of the SOI. (9) Dissolve the black adhesive to obtain individual electrodes. (10) Perform dry etching on the backside of the electrodes to release residual stress. **b** Release of residual stress by etching the backside of the MEAs. **c** Electrode structure, tip morphology (top), and actual MEA image (bottom). **d** Stress simulation results of electrodes with unrelieved residual stress (left) and with relieved residual stress (right)

**Fig. 2:**
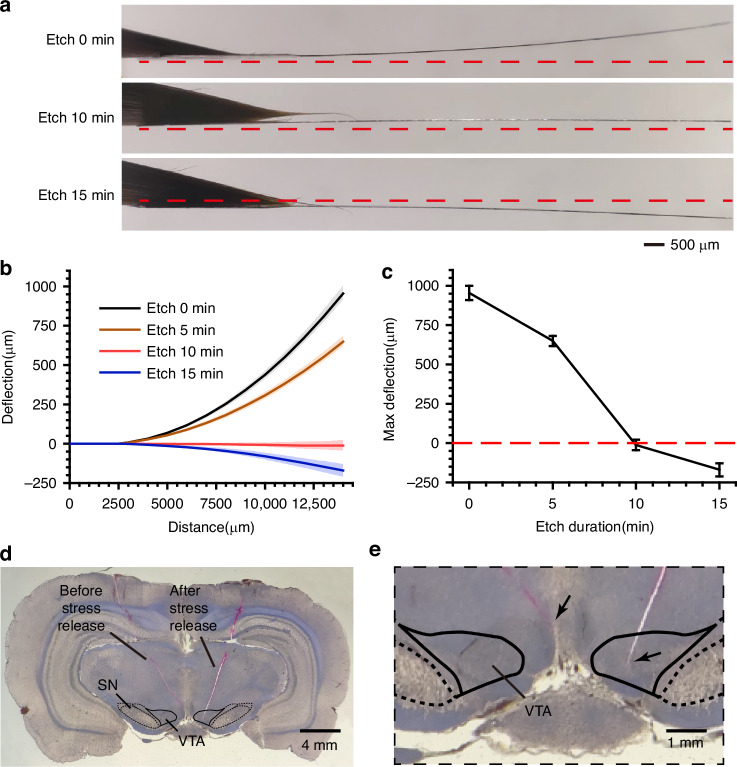
Characterization of residual stress-relieved MEAs and histological verification. **a** Photograph of the electrode taken from the side. The red dotted lines indicate the horizontal direction. **b**, **c** Electrode deflection and maximum deflection (deflection at the end of the electrode) for different etching durations. A positive deflection indicates that the electrode was bent toward the front. When etched for 10 minutes, the residual stress was relieved (max deflection = −11.8 ± 32.9 μm, mean ± SD, *n* = 5). **d**, **e** Histological verification. Micrograph of the coronal plane of the brain with 2 electrodes implanted simultaneously. The types of MEAs corresponding to the trajectories have been annotated. **e** Enlarged view of a portion of (**d**). The arrows in (**e**) indicate the electrode implantation sites

Electrode fabrication was based on standard MEMS technology (Fig. 1a). In brief, first, the silicon-on-insulator (SOI) wafer (25 μm Si/1 μm SiO_2_/350 μm Si) was cleaned via concentrated sulfuric acid (Fig. 1a (1)), and a 500 nm SiO_2_ film (the first insulating layer) was deposited on the SOI surface via thermal oxidation (Fig. 1a (2)). Thereafter, the metal layer (including detection sites, lead wires, and bonding pads; Cr 30 nm, Pt 250 nm) was formed through lithography, sputtering, and lift-off (Fig. 1a (3)). Next, 300 nm SiO_2_ and 500 nm Si_3_N_4_ were successively deposited as the second insulating layers via Plasma-Enhanced Chemical Vapor Deposition (PECVD) (Fig. 1a (4)). Si_3_N_4_ is used as a passivation layer to enhance the mechanical properties and insulation performance of the electrode surface^32–34^. Due to the gaps between the wires and sites on the metal layer, approximately 76.8% (on the electrode shank) of the area between the first insulating layer (SiO_2_) and the second insulating layer (SiO_2_/Si_3_N_4_) is in direct contact. The silicon oxide of the first insulating layer and that of the second insulating layer can jointly balance the stress of the silicon nitride film, maintaining the stability of the Si_3_N_4_ film. Reactive ion etching (RIE) was subsequently used to expose the detection sites and bonding pads and eliminate SiO_2_ from the bottom surface of the SOI (Fig. 1a (5)). The photoresist AZ4620 was spin-coated on the surface of the SOI, and the MEA shape was formed through lithography and deep silicon etching (Fig. 1a (6)). Finally, after applying the photoresist BN303 and black adhesive to protect the front of the SOI (Fig. 1a (7)), individual MEAs were obtained through wet etching (30% KOH, 80 °C) of the back of the SOI (Fig. 1a (8)), and the black adhesive with the negative photoresist developer was dissolved to obtain individual electrodes (Fig. 1a (9)). The fabricated electrode shank bent toward the front of the MEAs (Fig. 2a), indicating that the electrode was under compressive stress. To release the residual stress, a layer of photoresist was spin-coated on a silicon wafer. The front of the MEA was then manually attached to the photoresist, and RIE was used to etch the buried oxygen layer on the back of the MEA (Fig. 1b). The electrode structure, tip morphology and photograph of the residual stress relieved MEAs are shown in Fig. 1c. The electrodes are subsequently connected to the matching printed circuit board (PCB) through ultrasonic bonding, making them ready for implantation into the rat brain. The simulation results of etching for 0 minutes (initial status) and etching for 10 minutes (residual stress relieved) show that backside dry etching balances the stress distribution of the MEA, thereby enhancing its mechanical characteristics (Fig. 1d).

### Animals and surgery

We used four adult male Sprague‒Dawley (SD) rats (300‒330 g, ~8‒11 weeks old; Vital River Laboratory Animal Technology Co., Ltd.). All the rats were housed at a constant temperature (~22 °C) and humidity (~50%) and maintained on a 12-hour light/dark cycle. Initially, they had free access to food and water; then, they were subjected to restricted food (maintained at 85% ad libitum body weight). All experiments were performed with the permission of the Beijing Association on Laboratory Animal Care and were approved by the Institutional Animal Care and Use Committee at the Aerospace Information Research Institute, Chinese Academy of Science (AIRCAS).

**Fig. 3:**
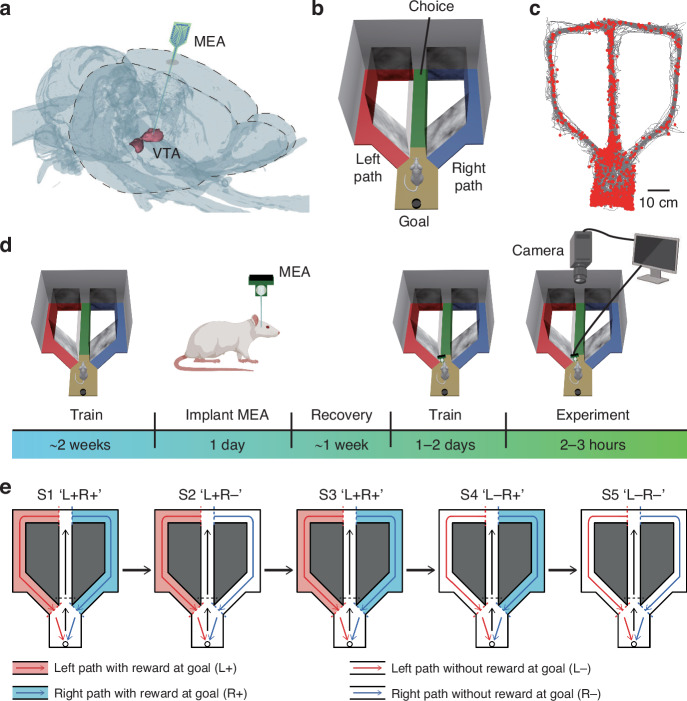
Electrophysiological recording and behavioral tasks. **a** A microelectrode array was implanted in the VTA of the left hemisphere. **b** Schematic diagram of the modified T-maze. The maze is fully enclosed; some of the sidewalls are not drawn, and all the sidewalls and the ground are black and opaque. The black well at the bottom is the goal position where the food reward can be dropped from a feeder above. The end of the middle arm (green) is the choice position, where the rat chooses to enter the left path (red) or the right path (blue). **c** Rat trajectories (gray lines) and spike locations (red dots) are plotted. **d** Schematic representation of the behavior, surgery, and electrophysiological activity recordings. **e** Diagram of the experiment, which consists of 5 sessions. Whether the food reward is dropped at the goal depends on the chosen path, and the color-filled arm indicates the correct path. L: left path; R: right path; ‘+’: reward; ‘−’: no reward

The surgical procedure was uniformly applied to all the rats. First, the rat was deeply anesthetized with 4–5% isoflurane, and its head was fixed on a stereotaxic apparatus. During the surgical procedures, the concentration of isoflurane was maintained between 0.8% and 1.8% to ensure anesthesia, and the depth of anesthesia of the rat was checked by pressing the tip of the tail or the foot. The hair of each rat was shaved, and the scalp was clipped to expose the skull. Five screws were drilled around the edge of the skull and connected to a metal wire used as a ground wire. A 3 mm square window was then drilled in the skull centered on the implant location (AP: −5.3 mm; ML: 0.85 mm, left hemisphere). The MEA with residual stress relief was slowly implanted through the window at a 15-degree angle into the VTA (DV: 8.4 mm from the dura level). By tilting the injection at 15 degrees, the implantation site bypasses the central large blood vessel. The ground of the MEA was connected to the ground wire. Finally, dental cement was used to secure the electrodes and protect the wound. Medical-grade Vaseline, which has good biocompatibility, is used to isolate the wound site of the implanted electrode from the dental cement during surgery. This not only aids in wound healing and sealing but also isolates the electrode from the mechanical influences of the dental cement. A schematic of MEA implantation in the VTA is shown in Fig. 3a. One rat was additionally implanted with an MEA (without residual stress relief) at the symmetrical implantation site in the right hemisphere (AP: 5.3 mm, ML: 0.85 mm) to compare the accuracy of the implantation position with and without residual stress relief (this rat did not participate in the behavioral task). After surgery, the rats received an intraperitoneal injection of 40,000 units of sodium penicillin to reduce the risk of bacterial infection.

### Behavioral task

As shown in Fig. 3b, the modified T-maze consists of a rest zone (yellow), a middle arm (green), a left path (red), and a right path (blue). A single trial began with the rat entering the middle arm, followed by a free choice of the left or right path at the choice position (i.e., the end of the middle arm; the left and right paths are completely symmetric). The rat then walked along the chosen path to the goal position until it entered the middle arm again. When the rat was in the rest zone, the left and right paths were blocked by the doors (next to the rest zone) such that the rat could only start along the middle path. Once the rat chose the left or right path, the door of the corresponding path was opened to allow the rat to return. Once the rat returned to the rest zone, the corresponding door was closed. Whether the rat received food reward (a small piece of peanut) at the goal depended on the chosen path. The food reward was delivered approximately 2 seconds after the rat reached the goal (if the rat chose the correct path).

As shown in Fig. 3d, before surgery, the rats were trained in the modified T-maze for approximately two weeks. During training, food reward was delivered regardless of whether the left path or the right path was selected. When the rat chose one path and then went to the other path or returned from the middle arm, no reward was given at the goal (error trial). Initially, the rats were trained for 20 to 40 trials per day, and they were later trained for 60 to 100 trials per day until they learned the experimental paradigm and the probability of error trial was less than 10%. After training, the rats had no preference for the left or right path in terms of behavior.

Five to seven days after surgical recovery, the rats were trained in the T-maze for 1 to 2 days before the experiment. The behavioral experiment was subsequently performed for 2‒3 hours. The paradigm of the experiment is shown in Fig. 3e. There are 5 consecutive sessions (S1 ‘L+R+’, S2 ‘L + R−’, S3 ‘L+R+’, S4 ‘L−R+’, S5 ‘L−R−’; L: left path, R: right path, ‘+’: reward, ‘−’: no reward, e.g., ‘L+R−’ means that the rat would receive food reward at the goal for choosing the left path and no reward for choosing the right path). Each session consisted of more than 20 trials (except for ‘L−R−’). Session switching did not involve any external sensory cues and only changed the reward probability corresponding to the path.

### Neuronal activity recording and behavioral tracking

The behaviors of the rats during the experiment as well as the neuronal activities in the VTA, were recorded synchronously. The MEA was connected to the neural signal detection instrument (AIRCAS-128, China)^35^ through a 16-channel headstage (custom-made, serving as the primary amplifier and filter of neural signals) and a light and sufficiently long wire to acquire the electrophysiological signals in the brain (sample rate of 30 kHz). A camera positioned above the T-maze recorded videos of the rats’ behaviors during the experiment. The trajectory of the rat (e.g., position, speed) was extracted offline from the videos via behavior analysis software (EthoVision XT 16, Noldus, China) at 30 Hz.

### Criteria for identifying neurons related to reward

We extracted the neural activity 2 seconds before and 4 seconds after the rat reached the goal in each trial and plotted the spike raster based on the outcome (reward or no reward) (e.g., Fig. 5a). The 6-second period was divided into 30 bins (200 ms per bin), after which the average firing rate of each bin was calculated and smoothed via a gaussian kernel^36^. After smoothing, the data were baseline corrected by subtracting the firing rate in each trail of each neuron from the mean firing rate from −2 s to −0.5 s for that trial (relative to the moment that the rat reached the goal)^11,37^. The plotted baseline-corrected neuronal firing rate curves are shown in Fig. 5a, b, d, e.

First, the firing rate after reaching the goal position must be significantly higher than the firing rate before reaching the goal position when a reward is present (paired t test). Second, the response to the outcome of neurons must be greater when a reward is present than when there is no reward (one-way ANOVA). Neuron responses to the outcome were parameterized as the Δ firing rate, which represents the difference between the average firing rate from 2 s to 3.5 s and the average firing rate from −2 s to −0.5 s (i.e., the average value of the baseline-corrected firing rate from 2 s to 3.5 s). A neuron is considered related to reward only if it meets both criteria simultaneously.

### Generation of spatial firing rate maps

The trajectory and outline of the T-maze were superimposed for display (Fig. S3a). By projecting each position to the nearest point on the centerline (total length ~198 cm) of the maze, the two-dimensional positions were transformed into linearized positions (Fig. S3b). Each spike was temporally associated with its nearest linearized location. The linear trajectory was divided into 99 bins (2 cm per bin). The occupancy time (the time spent in a bin) and spike counts in these bins were smoothed via a Gaussian kernel. Speed-filtered spikes and positions (speed>11 cm/s) were used for both one-dimensional (1D) and two-dimensional (2D) firing rate maps^38^. One-dimensional spatial firing rate maps (speed filtered) were calculated as the spike counts in each bin divided by the occupancy time in the corresponding bin. Two-dimensional firing rate maps (speed filtered) were constructed similarly but using a 1.5 × 1.5 cm bin. Moreover, the firing rate maps of each session were calculated separately, and for the 1D rate map, the left and right paths were also calculated separately.

### Determination of place fields

The speed-filtered 1D firing rate map was used for determining the place field, and the 2D firing map was plotted for illustration. A place field is defined as continuous bins meeting the following criteria^38–41^: (1) the firing rates of at least five continuous bins are more than 30% of the maximum firing rate across bins, (2) the average firing rate during the analysis period is >0.1 Hz, and (3) the peak firing rate in the place field is greater than 2 Hz.

### Statistical analysis

All the statistical analyses were performed via Python (Python 3.9) and ORIGIN (Origin Lab, USA). Unless otherwise stated, the data are presented as the mean ± SEM. The statistical significance within the same group was determined via paired t test. One-way ANOVA was used to compare the means between different groups, after which Tukey’s post hoc test was performed. Unless otherwise stated, *p* < 0.05 was used as the criterion for indicating a significant difference (*p < 0.05; **p < 0.01; ***p < 0.001).

## Results

### Residual stress relief of the MEAs

The VTA in the brain is deep and small, and it is close to the central blood vessel in the vertical direction. Therefore, many studies have implanted probes at tilted angles^42–44^. Because of these factors, accurately implanting the electrode is challenging, and a bending electrode shank makes it even more difficult. Therefore, we explored the method to relieve residual stress and thus reduce the curvature of the electrode.

The MEA exhibited different bending states depending on the duration of backside etching (Fig. 2a–c). As shown in Fig. 2b, c, when etched for 5 minutes, the residual stress of the electrode was partially released, and the maximum deflection of the electrode was reduced. When etched for 10 minutes, the buried oxide layer on the back of the MEA was properly etched (the thickness of the SiO_2_ film remaining was approximately 265 nm), and the maximum deflection was −11.8 ± 32.9 μm (mean ± SD, *n* = 5), indicating that the residual stress was relieved. When etched for 15 minutes, the buried oxygen layer on the back of the MEA was completely etched, and the electrode shank was bent toward the back, which means that the MEA was subjected to tensile stress. The etching depths at different etching times are provided in the Supplementary material. As shown in Fig. 2d, e, the electrode in which no residual stress was relieved (maximum deflection = 945.8 ± 31.5 μm, mean ± SD, *n* = 5) deviated from the VTA (left), whereas the electrode in which residual stress was relieved accurately reached the VTA (right). The histological results demonstrated that the residual stress relieved electrode improved the implantation accuracy.

Compared with previous studies, our electrodes exhibit lower curvatures (Table 1). Due to the structural similarity between Neuropixels^25,26^ and our electrodes, with both feature membrane structures on the upper and lower surfaces of a silicon substrate, the bottom layer being silicon dioxide and the top layer comprising silicon dioxide, metal layers, and silicon dioxide/silicon nitride layers, this method may also be applied to Neuropixels. Additionally, this approach can be extended to other MEAs with similar structures (e.g., electrodes that are longer or shorter, single-shank or multishank) to fabricate low-curvature electrodes.

**Table 1:** Comparison of different degrees of electrode bending

Electrode	Bending @ Electrode Shank Length
Neuropixels 1.0	≤100 μm @ 10 mm
Neuropixels 2.0	≤200 μm @ 10 mm
This work	≤43 μm @ 11 mm −11 ± 32.9 μm (mean ± SD)

### Behaviors and electrophysiological recording

After the electrode sites were modified with PtNPs, the electrode impedance at 1 kHz decreased from 1590.4 kΩ to 20.2 kΩ (Supplementary Fig. S1), and when the electrode was implanted, the rat began performing the behavioral experiment (Fig. 3). The behavioral result of a rat is shown in Fig. S2. The rat’s path choices were consistent with the probabilities of the reward corresponding to the path across 5 sessions. When the probability of receiving reward for the left and right paths was 1.0, the rat showed no preference for the left or right path (except for the third session, S3 ‘L+R+’). When the reward probability of one path was 1.0 and the reward probability of the other path was 0, the rat gradually preferred the path with the reward (S2 ‘L+R−’, S4 ‘L−R+’). When the reward probability corresponding to both paths was 0, the rat showed no preference for either path, and the number of error trials (in which goal-directed navigation was incorrectly performed) increased.

**Fig. 4:**
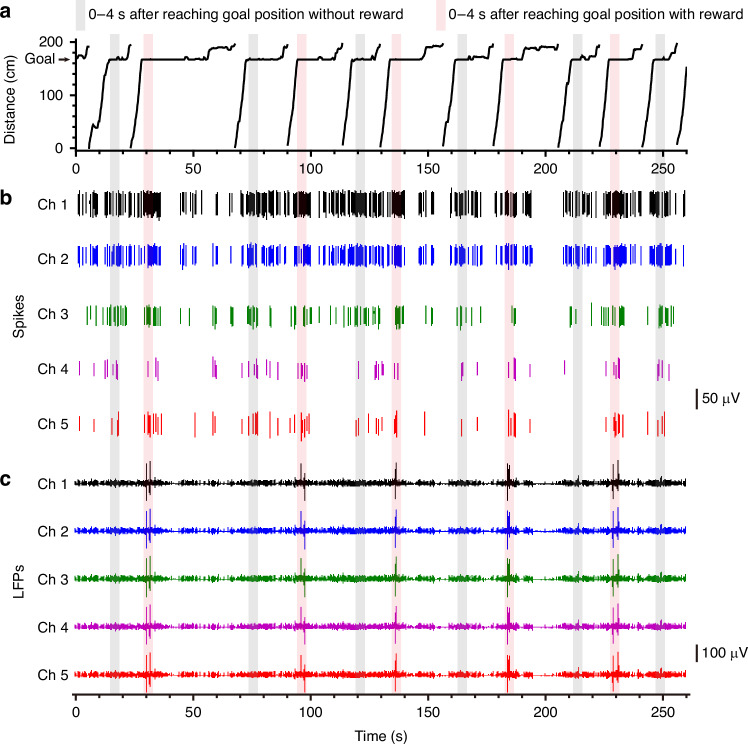
Behavioral performance and electrophysiological recordings. **a** Rat movements (distance from the start line, see Fig. S3) during several trials. **b** Recordings of spikes of a single unit in parts of the channels during 260 s of the ‘L + R−’ session (marked in **a** with the gray shaded window). Periods within 4 s after reaching the goal position were denoted by shaded windows (red, reward; gray, no reward). **c** Traces of LFPs corresponding to **b**

While recording the movement trajectory of each rat, we simultaneously recorded the electrophysiological activity of the VTA neurons via the low-curvature MEA. After high-pass filtering (>200 Hz) of the electrophysiological signal, spikes were extracted via a fixed threshold method. A total of 97 neurons from three rats were recorded. Figure 4b, c shows example spikes and local field potentials of VTA neurons recorded by the MEA during the experiment. As shown in Fig. 4b, the spikes in the red shaded windows (goal position with reward) are denser than those in the gray shaded windows (goal position without reward). In accordance with the firing activity of spikes, the amplitudes of local field potentials (LFPs) during the reward trials were much greater than those during the baseline and no-reward trials (Fig. 4c). Previous findings suggest that the LFPs of VTA neurons under prolonged (minutes to hours) excitatory stimulation (e.g., cocaine, morphine) have greater amplitudes than those in the control group (e.g., saline)^45,46^. Our results further suggest that the LFPs of VTA neurons can respond rapidly (within seconds or even less) to reward (excitatory stimulation).

### Neural activity related to reward

**Fig. 5:**
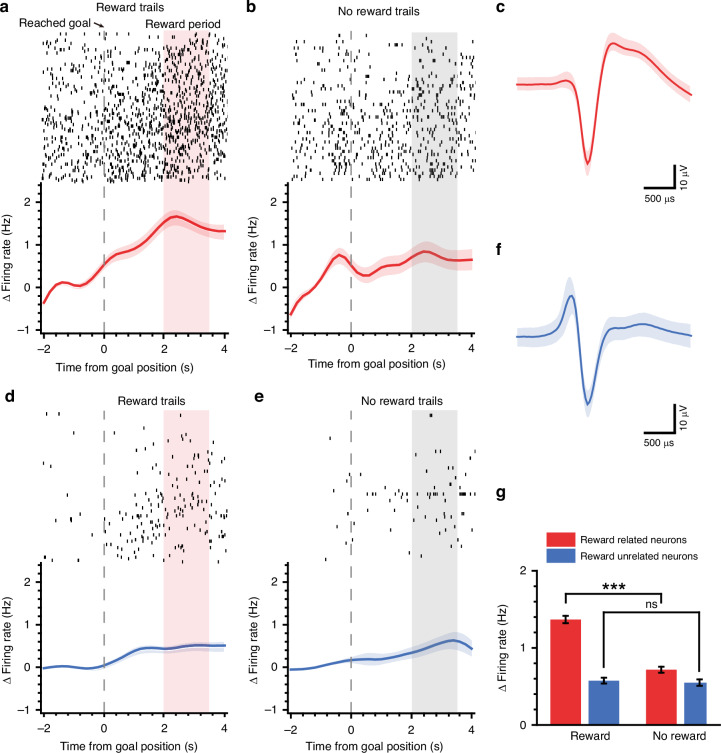
Neural activity related to reward. **a**–**c** An example neuron related to reward. **d**–**f** An example neuron unrelated to reward. **a**, **d** Neural activities when the rat received food reward at the goal position. The moment when the rat reaches the goal position is marked as time zero (dotted line), and the food reward is dropped at the goal approximately 2 seconds later. The spike raster across trials (each row indicates a trial) is plotted in the top panel. Evolution in time of the responses (i.e., Δ firing rate, baseline subtracted) to reward is plotted in the bottom panel. **b**, **e** Neural activities when the rat did not receive food reward at the goal position. **c**, **f** Spike waveform of an example neuron, mean ± SD. **g** Under the circumstances of receiving a reward and no reward at the goal, the response (i.e., the average firing rate during the shaded window in (**a**–**e**)) of different population neurons is plotted (red, reward-related neurons, *n* = 33; blue, reward-unrelated neurons, *n* = 64). One-way ANOVA test

As mentioned earlier, VTA neurons at the goal presented different activities between reward and no-reward situations. Hence, we investigated the response of VTA neurons to outcomes via one-way ANOVA (see Materials and Methods). A proportion of the neurons responded significantly differently to reward (*n* = 33, 34%). For neurons related to reward, the firing rate significantly increased during the reward trials compared with the period of 2 s before the rat reached the goal (paired t test) (an example neuron in Fig. 5a), which is in accordance with previous findings^47,48^. In contrast, there was no significant difference in the firing rate before and after the goal was reached for no-reward trials (Fig. 5b). The neural activity of an example neuron unrelated to reward is shown in Fig. 5d, e. The response during reward trials of population neurons related to reward (average firing rate during the 2–3.5 s duration of the reward-consuming period after reaching the goal) was significantly greater than that during the no-reward trials (Fig. 5g). These neural activities indicate the involvement of VTA neurons in reward coding.

### Remapping induced by reward

**Fig. 6:**
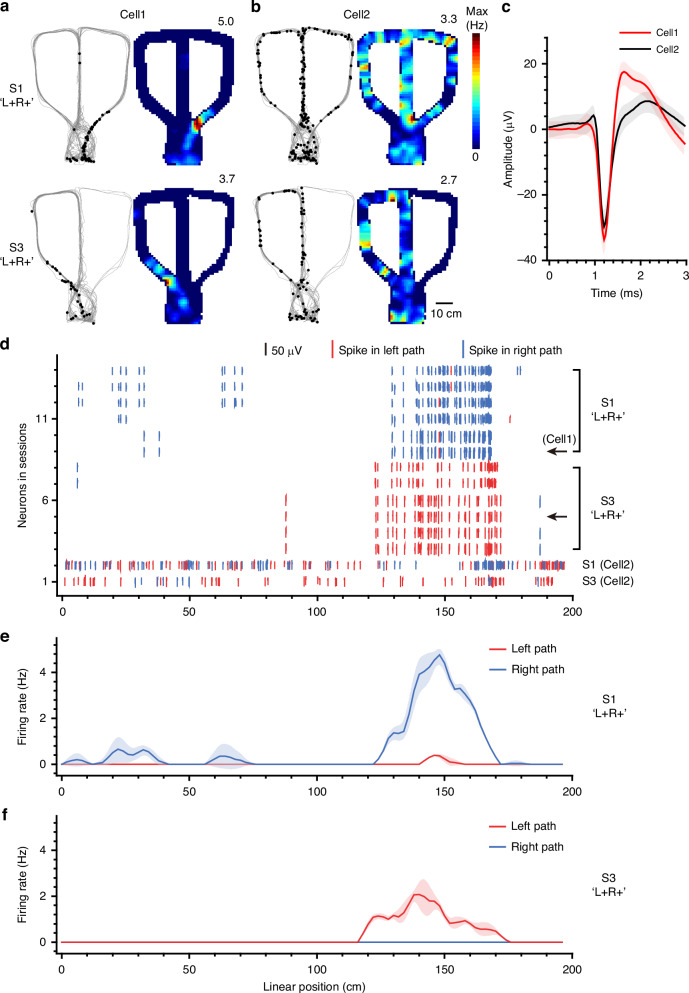
Remapping induced by reward. **a** An example neuron (Cell1) with place fields. Left panel, gray line, the rat’s trace; black dot, spike. Right panel, corresponding 2D firing rate map (the maximum firing rate is marked at the upper right corner). Top panel, session 1 (S1, ‘L+R+’); bottom panel, session 3 (S3, ‘L+R+’). **b** An example neuron (Cell2) without a place field. **c** Comparison of spike waveforms (mean ± SD) between Cell1 and Cell2. **d** Spikes of neurons with or without place fields in session 1 (S1, ‘L+R+’) and session 3 (S3, ‘L+R+’) are displayed in the spatial domain. **e** Evolution in the linear position of the firing rate (red line, left path; blue line, right path; mean ± SD) for neurons with a place field during S1 (‘L+R+’). **f** Same as (**e**) but for S3 (‘L+R+’)

During the goal-directed navigation experiment, the rat succeeded in learning the change in the relationship between path and outcome (Fig. S2). The behavioral results indicate that the rat can learn hidden cues contained in environmental changes without external sensory cues^49^ and even without separate goal locations for different paths^39^. Recent studies have shown that the VTA is related not only to reward and cues^2,8,20^ but also to environmental variables such as position, speed, and distance^13,50^. Therefore, we analyzed neural activities in the spatial domain across five sessions. Since the path of the T-maze was relatively narrow (only 8 cm in width), we converted the two-dimensional positions to one-dimensional positions first, and the data of the trials in each session were then used to generate one-dimensional spatial firing rate maps of the left and right paths separately^39,40^ (see Materials and Methods). The 2D firing rate maps are plotted in Fig. 6a, b. An example neuron (Cell 1) with a place field is shown in Fig. 6a, b displays another example neuron (Cell 2) without a place field. A total of 12 neurons exhibited place fields. All of them had a place field in session 1 (S1, ‘L+R+’), but only 9 neurons had a place field in session 3 (S3, ‘L+R+’). The spikes in S1 and S3 are shown in the spatial domain (Fig. 6d), and the average firing rate curves of neurons with place fields during S1 and S3 are plotted in Fig. 6e, f.

In session 1 (‘L+R+’), as the initial status, the place field was located at a position slightly before the goal position in the right path (Fig. 6a, e). In session 2 (‘L+R−’), there was no reward at the goal position when the rat chose the right path, and the place fields disappeared (not shown). By session 3 (‘L+R+’), the probabilities of receiving food reward by choosing the left and right paths were both 1 (same as that of S1‘L+R+’). The place field was formed in the left path (Fig. 6a, f). The location of the new place field was slightly before the goal position in the left path (symmetric with the location of the place field in the first session). In contrast, the firing rate of the right path was low. This corresponds to the behavioral result that the rat had a preference for the left path in session 3 (‘L+R+’) (Fig. S2). The peak firing rate of the place field decreased significantly (from 4.79 ± 0.36 Hz in S1 to 2.39 ± 0.36 Hz in S3, mean ± SD, one-way ANOVA, *p* < 0.001), potentially indicating that neurons could complete similar learning processes with lower energy consumption. In session 4 (‘L−R+’), the place field in the left path disappeared, and the neural activity in the left path became dispersed. In the last session (‘L−R−’), the neural activity in the left and right paths was severely reduced. Based on the above phenomenon, the disappearance and reconstruction of the place fields correspond to the change in the relationship between path and outcome.

## Discussion

Here, we designed and fabricated low-curvature microelectrode arrays with PtNPs modified on the site surface. The electrode impedance (20 kΩ) is significantly lower than that of TiN-coated electrodes (Neuropixels) (149 kΩ)^25^ and TiW/Pt electrodes (81 kΩ)^51^. Using our self-developed electrode, we recorded and analyzed the electrophysiological activity of VTA neurons during goal-directed navigation in a freely chosen T-maze with a changing path‒outcome relationship.

Electrode fabrication is based on the SOI and involves multiple film deposition, patterning, etching, and finally wet corrosion release. The residual stress in the fabricated MEAs is due mainly to the residual stress of the SOI itself^52^ and the thermal stress generated by the high-temperature environment in the deposition processes of the multilayer film^23,24^. Due to the numerous processes and difficulties in controlling the production environment and equipment state, residual stress relief after electrode fabrication is advised. Compared with the corrosion of SiO_2_ via HF buffer^53^, backside dry etching can precisely control the etching depth through the etching duration and has advantages in terms of operational safety and convenience. Additionally, this method of precisely controlling the deflection by changing the residual stress can produce electrodes with controlled curvature, which have potential applications. Moreover, we established a simplified mechanical simulation model for the electrode before and after residual stress relief. Residual stress relief not only solved the problem of electrode bending but also optimized the stress distribution inside the electrode (reducing the concentrated stress caused by the rapid reduction in the width of the electrode at the junction of the shank and pad). Histology verified that the residual stress relieved electrode has greater spatial implantation accuracy. The modified PtNPs on the surface of the electrode sites reduce the electrode impedance and thus improve the signal-to-noise ratio. For flexible electrodes^54,55^, bending has little impact on implantation accuracy. The implantation of flexible electrodes is usually facilitated by temporarily hardening the electrode (e.g., coating with PEG^56^, PVA/PLGA^28^) or using rigid materials (such as glass capillaries and ultrathin metal insertion needles^57^) to guide implantation. Consequently, the problem of electrode bending is cleverly circumvented by using external rigid materials. Once implanted in the brain, the electrode regains its flexibility, and the brain tissue restricts the position of the flexible electrode, allowing it to remain in the target location. If the electrode bends, the residual stress may still be present. The impact of such bending has not yet been documented in the literature and may warrant further investigation.

The ventral tegmental area is famous for the RPE signal, in which dopamine neurons increase their firing rate when the unexpected reward is encountered and decrease when a predicted reward is omitted^2,4,7^. Many studies have investigated the relationship between dopamine neurons and varying levels of reward^8^. The greater the amount (or higher probability) of reward is, the greater the response of dopamine neurons. These experiments typically include a conditioned stimulus (e.g., gas, sound) representing the presence or absence of reward. In the present study, we omitted this conditioned stimulus and used the path as the determinant for the presence or absence of reward at the goal position. Using the arrival of the rat at the goal location as the starting point of the event, we analyzed the neural activity of VTA neurons before and after this event. The results revealed that a subset of neurons in the VTA were involved in the process of encoding reward. The firing rate increased after the rat reached the goal during the reward trials, and the response during the reward trials was significantly greater than that during no- reward trials. In addition to reward coding, VTA neurons are also involved in encoding other variables, such as kinematic and environmental factors^13,50^. During goal-directed navigation, the rat must continuously learn the changing path‒outcome relationship. After analyzing one-dimensional firing rate maps, we identified 12 neurons with place fields. These place fields disappeared and were reformed in accordance with the changes in reward conditions. This finding indicates that VTA neurons are also involved in the encoding of spatial position and that this encoding relationship can be influenced by reward. The locations of the place fields were before the goal position, which may suggest the prediction of the reward or the goal value. Previous studies have identified the neuronal projection from the VTA to the hippocampus^58^ and its modulating effect on spatial memory, learning, and other aspects of hippocampal fuction^48,59–62^. Changes in spatial firing rate maps of VTA neurons under different reward conditions may also promote the generation and stability of place fields in hippocampal place cells, thereby facilitating goal-directed navigation. Furthermore, whether the remapping of VTA neurons induced by reward is generated by the VTA itself or derived from other brain regions remains to be investigated. Since reward conditions are tied to specific paths and spatial navigation is associated primarily with brain regions such as the hippocampus, entorhinal cortex, and prefrontal cortex^1^ and given the complex neural projections of the VTA^17^, these spatial firing characteristics are likely the result of combined inputs from multiple brain regions (spatial position encoding) and VTA neurons (value assessment).

## Conclusions

In the present study, we designed and fabricated low-curvature MEAs for the VTA. The maximum deflection of the low-curvature MEAs obtained via backside dry etching was reduced from 945.8 ± 31.5 μm to −11.8 ± 32.9 μm, which effectively improved the precision of electrode implantation. Neuronal activity in the VTA was recorded during goal-directed navigation in a modified T-maze. Notably, 34% (*n* = 33) of the neurons in the VTA responded to the reward. The firing rate during the reward-consuming period in the reward trials was significantly greater than that in the no-reward trials, and the amplitude of LFPs was greater than that in the baseline and no-reward trials. Furthermore, 12% (*n* = 12) of the neurons presented spatial firing characteristics, and the place fields remapped correspondingly with changes in reward conditions. These results show that the VTA is involved not only in reward coding but also in spatial coding. This study provides a technical means for fabricating low-curvature MEAs and lays the foundation for further understanding the role of the VTA in goal-directed navigation.

## Supplementary information

Supplementary information
