# Supplementary material for: Neuronal activity in the ventral tegmental area during goal-directed navigation recorded by low-curvature microelectrode arrays
Source: Microsyst Nanoeng. 2024 Oct 14;10:145. doi: 10.1038/s41378-024-00778-2 (PMC11471829; doi:10.1038/s41378-024-00778-2)
Supplement: Supplementary file 1 — Supplementary information [file 41378_2024_778_MOESM1_ESM.docx]

Neuronal activity in the ventral tegmental area during goal-directed navigation recorded by low-curvature microelectrode arrays

*Wei Xu^1,2^, Mixia Wang^1,2^, Gucheng Yang^1,2^, Fan Mo^1,2^, Yaoyao Liu^1,2^, Jin Shan^1,2^,Luyi Jing^1,2^, Ming Li^1,2^, Juntao Liu^1,2^,Shiya Lv^1,2^, Yiming Duan^1,2^, Meiqi Han^1,2^, Zhaojie Xu^1,2^*, Yilin Song^1,2^*, Xinxia Cai^1,2^**

1 State Key Laboratory of Transducer Technology, Aerospace Information Research Institute, Chinese Academy of Sciences, Beijing 100190, China

2 School of Electronic, Electrical and Communication Engineering, University of Chinese Academy of Sciences, Beijing 100049, China

* Correspondence: Zhaojie Xu(xuzj@aircas.ac.cn), Yilin Song (ylsong@mail.ie.ac.cn) or Xinxia Cai (xxcai@mail.ie.ac.cn)

1. Supplementary Information
2. Morphology and electrical performance of MEAs

In this study, electrode sites were modified with platinum nanoparticles (PtNPs). The electrode tips under light microscopy are shown in Fig. S1a, and all sites were modified with black PtNPs. Scanning electron microscopy (SEM) reveals the rough and porous structures of PtNPs on the surface of the electrode site at the microscopic scale (Fig. S1b, c), which can increase the specific surface area of the site and thus improve the conductivity. The electrical characteristics of the electrode sites were evaluated by cyclic voltammetry (CV) from -0.6 to 0.8V(Fig. S1d) and electrochemical impedance spectroscopy (EIS) from 1 Hz to 1 MHz in PBS (Fig. S1e, f). The characterization results indicate that the electrode's charge storage capacity (CSC) increased from 8.27 mC/cm² to 96.76 mC/cm². The impedance of the electrode modified with PtNPs is reduced compared to that of the bare electrode. At the typical frequency of 1 kHz, the average impedance of electrode detection sites is significantly reduced from 1590.4 ± 263.1 kΩ (bare) to 20.2 ± 3.4 kΩ (PtNPs modified) (one-way ANOVA, p<0.001), which is beneficial to decrease the thermal noise and thus improve SNR (signal to noise ratio). And the phase angle reduced from -59° to -28°.


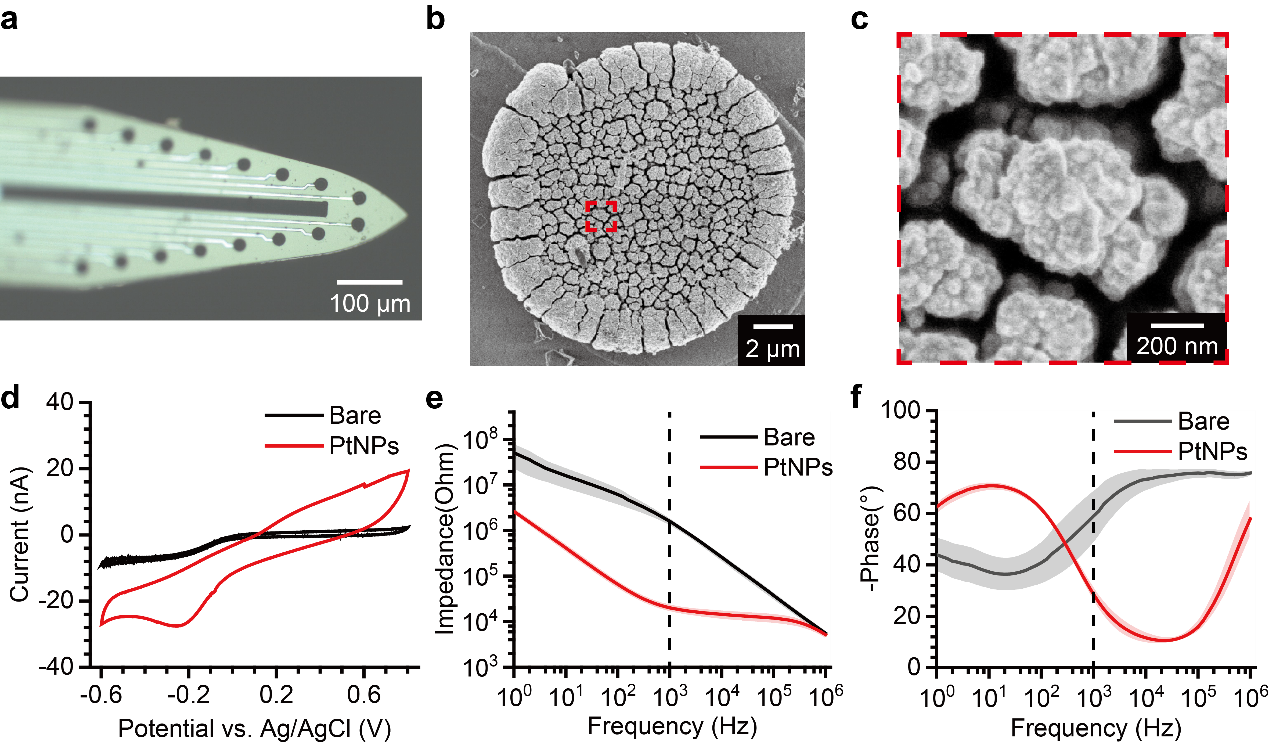


**Fig. S1 Electrochemical modification and** **characterization of electrode sites. (a)** Photo of PtNPs modified MEA. **(b, c)** SEM images of PtNPs modified electrode site in different scales (**b**, 2 μm; **c**, 200 nm). **(d)** The cyclic voltammetry (CV) curves from -0.6 V to 0.8 V. **(e, f)** The average impedance (e) and phase (f) of the electrode at different frequencies from 1 Hz to 1 MHz (n = 16 recording sites).

1. Etch depth evolution

Due to the small area of the electrode, the etch depth measurement cannot be performed using an ellipsometer. Instead, a step profiler (Alpha-Step D-300, KLA) was used for detection. The results are as follows:

For an etch time of 5 minutes, the etch depth is approximately 452.55 ± 65.53 nm.

For an etch time of 10 minutes, the etch depth is approximately 884.94 ± 52.52 nm.

For an etch time of 15 minutes, the etch depth is approximately 1220.09 ± 158.291 nm (mean ± SD).

The average etch rate is approximately 88.49 nm per minute.

However, since the electrode is curved, the SiO2 thin film on the backside of the electrode is also curved, and the measured values only represent the etch depth near the measurement point. Other positions may have different depths. Therefore, using etch time provides a more objective representation of the etching process. As a result, the article uses etch time as the characterization parameter for the etching extent.


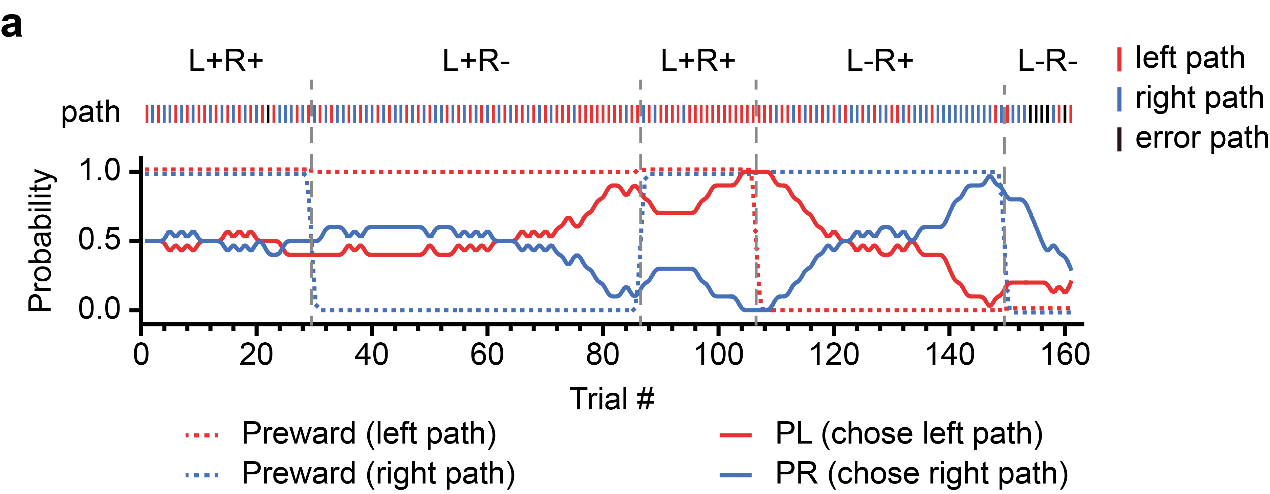


**Fig. S2 Behavior choice result.** An example of the rat’s choice behavior. The top panel represents the choices of the rat across 5 sessions (marked at the top). Vertical lines denote the chosen path (red, left path; blue, right path; black, error path, rat choose more than one path or return from the middle arm). The probability of choosing the left path (PL, red line) and the right path (PR, blue line) is plotted (moving average of 10 trials) on the bottom panel. Dotted lines denote the probability of getting food reward at the goal position under the chosen path condition (Preward. Red dotted line, left path; blue dotted line, right path).


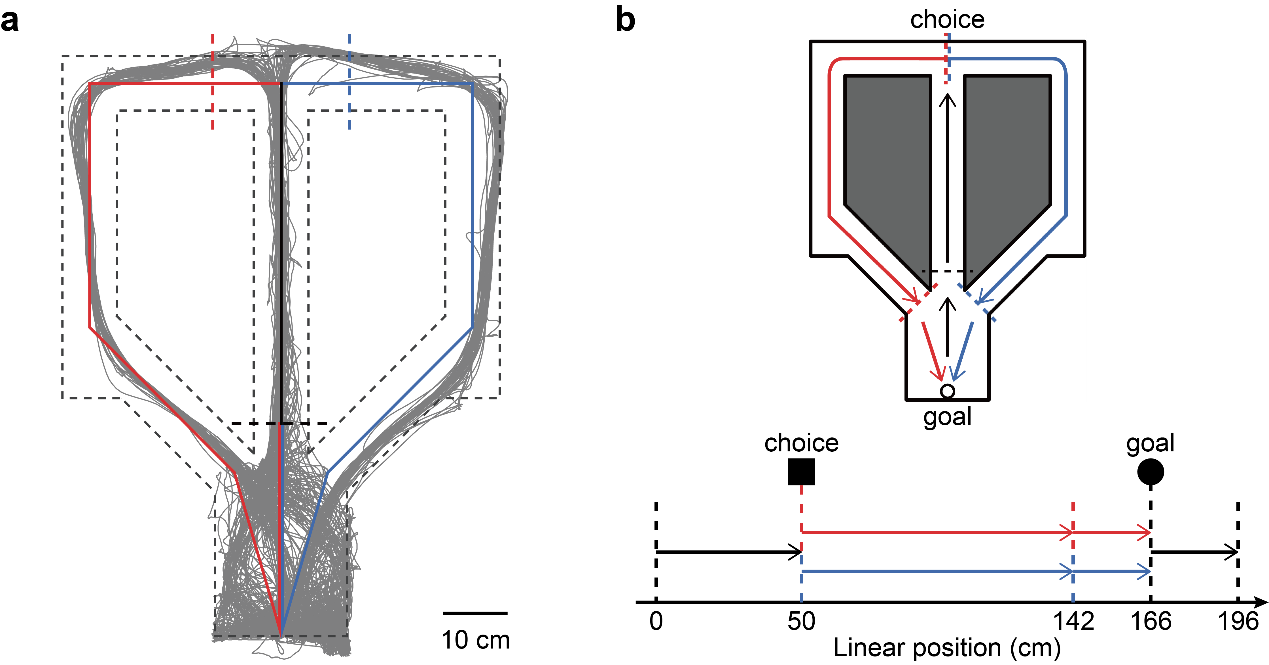


**Fig. S3 Trajectory processing.** **(a)** The trajectory of a rat with the outline of the modified T-maze. The line denotes the center line of the T-maze (red, left path; blue, right path; black, middle arm). All positions of the trajectory are projected onto the center line to calculate the linearized position. The black dotted line at the bottom of the middle arm indicates the start line of one trial. The dotted line at the top indicates the choice of the chosen path (red, left path; blue, right path). **(b)** Schematic diagram of the linearized trajectory.
